# Protein-based identification of quantitative trait loci associated with malignant transformation in two HER2+ cellular models of breast cancer

**DOI:** 10.1186/1477-5956-10-11

**Published:** 2012-02-22

**Authors:** Yogesh M Kulkarni, David J Klinke

**Affiliations:** 1 Department of Chemical Engineering, College of Engineering and Mineral Resources, West Virginia University, Morgantown, WV 26506, USA; 2 Department of Microbiology, Immunology and Cell Biology and Mary Babb Randolph Cancer Center, School of Medicine, West Virginia University, Morgantown, WV 26506, USA

**Keywords:** MALDI-TOF MS, Proteomics, Breast cancer, Malignant transformation, Two dimensional gel electrophoresis, Ingenuity pathway analysis

## Abstract

**Background:**

A contemporary view of the cancer genome reveals extensive rearrangement compared to normal cells. Yet how these genetic alterations translate into specific proteomic changes that underpin acquiring the hallmarks of cancer remains unresolved. The objectives of this study were to quantify alterations in protein expression in two HER2+ cellular models of breast cancer and to infer differentially regulated signaling pathways in these models associated with the hallmarks of cancer.

**Results:**

A proteomic workflow was used to identify proteins in two HER2 positive tumorigenic cell lines (BT474 and SKBR3) that were differentially expressed relative to a normal human mammary epithelial cell line (184A1). A total of 64 (BT474-184A1) and 69 (SKBR3-184A1) proteins were uniquely identified that were differentially expressed by at least 1.5-fold. Pathway inference tools were used to interpret these proteins in terms of functionally enriched pathways in the tumor cell lines. We observed "protein ubiquitination" and "apoptosis signaling" pathways were both enriched in the two breast cancer models while "IGF signaling" and "cell motility" pathways were enriched in BT474 and "amino acid metabolism" were enriched in the SKBR3 cell line.

**Conclusion:**

While "protein ubiquitination" and "apoptosis signaling" pathways were common to both the cell lines, the observed patterns of protein expression suggest that the evasion of apoptosis in each tumorigenic cell line occurs via different mechanisms. Evidently, apoptosis is regulated in BT474 via down regulation of Bid and in SKBR3 via up regulation of Calpain-11 as compared to 184A1.

## Background

Cancer cells exhibit a number of common traits that differentiate themselves from normal cells, including sustained proliferative signaling, resisting cell death, and evading growth suppressors [1]. Acquiring one of the hallmarks of cancer is associated with activating/deactivating particular genes, called oncogenes/tumor suppressors. The focus on identifying specific oncogenes or tumor suppressors that drive malignant transformation embodies the genomic era in cancer research [2]. However, a series of recent developments challenge this oncogene paradigm. Next generation sequencing efforts have revealed that, instead of a small number of genetic alteration associated with malignant transformation [3], genomic landscapes are extensively modified in solid tumors [4,5]. These massive rearrangements suggest that there might not be single driver mutations, but that the quantitative alterations in cellular traits associated with malignant transformation is distributed among multiple genetic loci. These quantitative trait loci (QTL) manifest themselves by altering the flow of intracellular information among signaling pathways [6]. In addition, evolutionary theory suggests that cancer cells are alternative solutions to a multivariate optimization problem where the tumor microenvironment provides the selective landscape [7]. Collectively, these findings suggest a more global approach towards identifying the molecular alterations associated with oncogenic transformation.

Gene microarray platforms provide tremendous breadth in surveying the gene expression landscape within a cellular system. However, relating gene expression with the specific role that the corresponding protein plays in a signaling pathway is complicated by post-transcriptional control of protein expression [8] and tight regulation of protein activity [9]. In contrast to genomic-based assays, proteomics provides an attractive platform to profile this regulatory layer of protein activity and differences in the level of protein expression [10]. Similar to QTL analysis [11], differences in proteomic patterns across biological systems that exhibit quantitative differences in traits provide an unbiased perspective to identify molecular mechanism that underpin these differences in phenotype. In contrast to QTL analysis, the direct relationship between proteomic profiles and cellular traits implies that a smaller sample size can still yield meaningful insight. High-throughput quantitative proteomic analysis has previously been used to identify differentially expressed proteins and pathways associated with breast tumor phenotypes [12]. Also networks derived from the differences in expression of key specific biochemical molecules between normal and transformed hepatocytes uncovered profound differences in the immune response between these cells [13]. More generally, knowledge of molecular mechanisms that associate with cellular traits may lead to new therapeutic strategies or new fundamental understanding of the corresponding signaling pathways. Despite this promise, the proteomic loci, as a manifestation of underlying genetic alterations that are associated with oncogenic transformation in breast cancer, remain unclear.

Breast cancer is a clinically heterogeneous disease with a variety of distinct subgroups of tumors endowed with different phenotypes and clinical outcomes. Using profiles of gene expression, breast cancers are divided into five major subtypes: triple-negative (ER-/PR-/HER2-), luminal A (ER/PR+,HER2-), luminal B (ER/PR/HER2+), HER2+/ER-, and normal breast-like (ER/PR/HER2-, CK5/6,HER1+) [14-16]. Clinical presentation is distributed among the breast cancer subtypes: 68% were luminal A, 9.5% were luminal B, 9.5% were HER2+/ER- and 13% were triple-negative and normal breast-like [17,18]. Particular subtypes also express unique patterns of proteins. For instance, HER2 expression is more common in HER2+/ER- and luminal B subtypes. Ki-67 and TP53 expression is rarely associated with luminal A subtype as compared to others. While these subtypes are defined to help tailor treatment options, it is unclear what pathway alterations occur in concert with ER/PR/HER2 amplification. In this study, our objective was to identify proteins that are differentially expressed in two HER2+ phenotypes of breast cancer (BT474 and SKBR3) as compared to a cell line that is reflective of normal mammary epithelium (184A1) using gel-based proteomics. Differences in protein expression were then analyzed using systems biology tools to identify the functionally enriched pathways. Immunoblotting was used to validate the patterns of protein expression observed using a proteomics workflow. In summary, we found that differentially expressed proteins in BT474 were overrepresented by proteins involved in cell proliferation and those in SKBR3 were overrepresented by proteins involved in amino acid metabolism. Differential protein expression also suggests that apoptosis signaling, a functionally enriched pathway that is inhibited in both the tumor phenotypes might have independent mechanisms unique to each tumor phenotype. In BT474, apoptosis is regulated by under-expression of Bid in comparison with 184A1; whereas, it occurs in SKBR3 by over-expression of Calpain-11 as compared to 184A1.

## Results

### Identifying proteins differentially expressed in breast tumor cell lines

#### 2DE and image analysis

**Figure 1:**
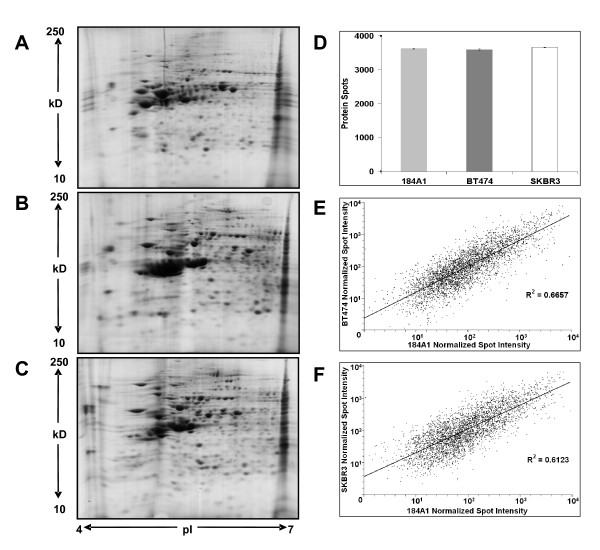
**Representative 2-D proteomic profiles of cell lines (A) 184A1, (B) BT474 and (C) SKBR3**. The first dimension was resolved on IPG strip 4-7, 7 cm. The second dimension is a 12% SDS-PAGE spanning molecular weight region 10-250 kDa, stained with coomassie blue and scanned using Typhoon 9400 scanner. (D) Quantitative image analysis using Ludesi REDFIN reveals the gel reproducibility and protein loading with no significant difference in the number of identified protein spots on the gel replicates across each cell line. Error bars represent S.E.M. Scatter plots of average normalized intensities are plotted on a logarithmic scale for matching protein spots showing the dynamic range of spot detection and correlation for the normal cell line 184A1 versus (E) BT474 and (F) SKBR3.

**Figure 2:**
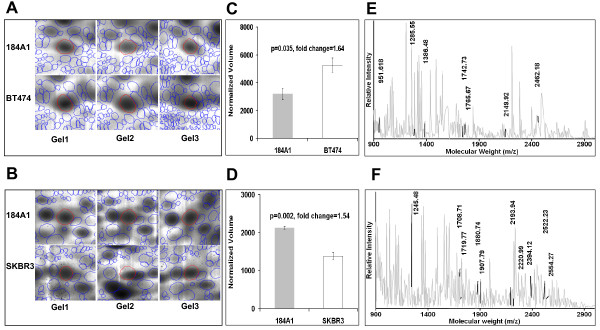
**Montage showing differential expression of (A) Rnase11 and (B) Hibadh on each gel across both comparisons (identified spot border is in red and neighboring spot borders are in blue)**. Densitometric quantitation of the normalized volume of the spot on each gel with the associated fold-change and corresponding *p*-value is shown for (C) Rnase 11 and (D) Hibadh. Mass spectra for each peptide digest was acquired between mass values of 800 and 3000, deisotoped using PLGS2.1 and submitted for peptide mass fingerprinting. The mass spectra is shown for (D) Rnase11 and (E) Hibadh with the peptide mass values that contributed towards successful identification of the protein indicated in bold and numbered on the spectrum.

Representative pattern of cellular proteome obtained in the pH range 4-7 after 2DE of total cellular extracts is shown in Figure 1 with more than 3,500 unique protein spots resolved in each cell line (Figure 1D). The proteomic pattern for each cell line was highly reproducible among biological replicates (Additional file 1: Figure S1) with a dynamic range spanning four orders of magnitude and a strong correlation between normalized intensities for matching spots (Figure 1E and 1F). Considering that there may be some basal differences in protein expression among these cell lines, a protein was considered differentially expressed if it was deregulated by a factor of at least 1.5-fold. Comparing the relative spot intensities in the 184A1 to BT474 cell lines revealed a total of 329 spots that showed a significant (*p *< 0.05) difference in expression by at least 1.5-fold. Similar comparison in 184A1 and SKBR3 proteomic profiles revealed 265 spots to have a significant (*p *< 0.05) expression change by at least 1.5-fold. Out of these, 110 well-resolved spots were selected in cell lines 184A1 and BT474 and 109 well-resolved spots were selected in cell lines 184A1 and SKBR3. An example of a well-resolved protein spot with its presence in each gel across both the cell lines is shown for 184A1-BT474 (Figure 2A) and 184A1-SKBR3 (Figure 2B) comparisons. The normalized volume of the spot in each cell line with the fold-change and significance is shown for 184A1-BT474 (Figure 2C) and 184A1-SKBR3 (Figure 2D) respectively.

#### Peptide mass fingerprinting

**Figure 3:**
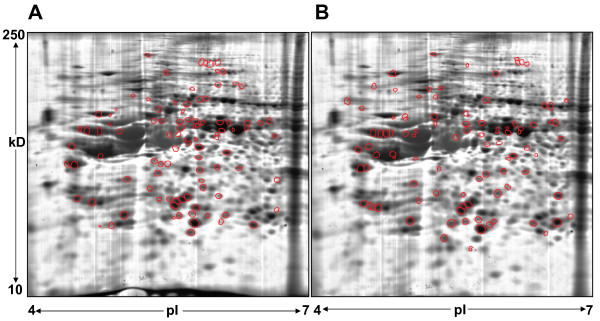
**Proteome map for 184A1 with red circles indicating protein spots differentially expressed by at least 1.5-fold (*p *< 0.05) that were identified in (A) BT474 and (B) SKBR3**. Identified proteins are shown in Additional File 2: Table S1 (184A1-BT474) and Table S2 (184A1-SKBR3).

The selected protein spots were excised, in-gel digested by trypsin and analyzed by MALDI-TOF MS to generate a peptide mass fingerprint. The resulting peptide mass fingerprints were queried against the Swiss-Prot human database using Mascot as a primary database search algorithm. Expasy Aldente was used as a complementary algorithm for additional confirmation to reduce the possibility of false positive identification (Additional file 2: Table S1 and Additional file 3: Table S2). Agreement between the apparent M_r _and pI observed on the 2-D gel and the theoretical values of the identified proteins provided additional support for positive identification. As an example, peptide mass fingerprints with the tryptic peptide mass values that contributed towards protein identification are shown for Probable ribonuclease-11 (RNASE11) in 184A1-BT474 and 3-hydroxy isoburate dehydrogenase (HIBADH) in 184A1-SKBR3 in Figure 2. From 110 picked spots, 96 differentially expressed proteins were identified in the 184A1-BT474 comparison. For the 184A1-SKBR3 comparison, 94 proteins were identified from 109 picked spots. These differentially expressed proteins that were identified in BT474 (Figure 3A) and in SKBR3 (Figure 3B) are encircled red on the proteome map of 184A1. Out of these, 64 proteins were unique to the 184A1-BT474 dataset and 69 proteins were unique to the 184A1-SKBR3 dataset. Some of the identified proteins may exist in multiple forms, as they were associated with multiple protein spots. It is unclear as to whether these proteins exist in multiple forms due to biological differences or processing influences such as carbamylation, a common modification when using urea buffer, that causes shifts in the isoelectric point of the protein. Different forms of keratins, a common source of contamination in this method of identifying proteins by in-gel digestion and PMF, also formed a small subset of identified proteins and were excluded from all subsequent analysis. More than 95% of the spots had sequence coverage exceeding 25%. These differentially expressed proteins (Table 1) formed the dataset for pathway analysis and network generation.

**Table 1:** List of unique proteins that were differentially expressed and formed the dataset for IPA analysis are listed along with the associated fold-change and *p*-value.

BT-474 versus 184A1				SKBR3 versus 184A1			
**Gene Symbol**	**Fold change^a^**	**Gene Symbol**	**Fold Change^a^**	**Gene Symbol**	**Fold change^a^**	**Gene Symbol**	**Fold change^a^**

ACTB	1.9 ± 0.1	LYN	1.62	AKT2	-3.14	LMNA	-2.6

ACTN4	-3.54	MCF2L	2	ALDH2	-1.72	LMNB1	1.98

ACTRT1	-3.75	NDE1	2.33	ANXA5	-6.5	MAP3K7	-4.95

ADSL	-2.58	P4HB	2.9 ± 1.1	ANXA6	1.73	MPP2	2.37

ALCAM	-21.5	PCYT1B	-3.45	APOA1	-2.71	MRPS35	5.18

ALDH2	-2.63	PDE4D	2.45	ATP5B	3.03	NME5	-6.4

ANXA5	-20.5 ± 141	PDE4D	-2.94	ATP5B	-3.2 ± 0.6	NME7	-6.4

ANXA8L2	-4.24	PHB	-2	BPNT1	-2.59	NQO2	-5.83

BCAR3	4.25	PIH1D1	-2.46	CAPN11	3.52	OPA1	-3.06

BID	-3.19	PMM2	2.74	CMPK1	-2.56	P4HB	2.55

C10orf88	-3.28	POLR3E	-2.12	CTNNA3	-3.46	PAF1	-1.97

CAPZB	-2.05	PPFIBP1	2.23	EEF2K	6.03	POLI	-2.11

CDKN2A	3.22	PPM1B	2.99	EIF2S1	-3.89	PPFIBP1	4.18

CSK	-9.11	PRDX2	-5.03	EIF2S2	4.32	PPP1CC	8.87

DPYSL4	1.66	PRPSAP2	-2.87	EIF3I	-3.89	PRDX6	3.21

EEFIG	-2.73	PSMB1	1.76	EVC	10.12	PRKAR2A	-19.47

EIF2B3	-3.95	PSMC2	-5.3 ± 2.5	FAM102B	2.08	PRPS1	-5.46

FSTL1	-7.6	PSMC4	-7.19	GDI2	-2.31	PRPSAP2	-2.31

GNA14	-3.1	PSME1	-2.2 ± 0.6	GNB1L	-6.1	PSMA3	-3.25

GSTP1	-45.37	PTER	-6.49	GOLGA8F	4.17	PSMB8	-3.7

HIBADH	-2.86	RAB27B	-1.83	GRK4	-2.8 ± 0.4	RAB37	-2.17

HORMAD1	-1.63	RAB37	-2.9 ± 0.4	GSTP1	-22.92	RABIL1	-3.01

HSPA1L	-3.53	RAB3A	3.45	GTF3C4	3.69	RMND1	-3.13

HSPA5	2.21	RGN	-1.62	HIBADH	-1.54	RNASE11	-9.38

HSPA9	5.0 ± 3.2	RNASE11	1.64	HNRNPF	2.63	SEMG2	-5.53

HSPB1	-2.48	RNPEPL1	-1.93	HSP90B1	1.57	SEPT1	-3.58

HSPD1	3.4 ± 0.9	RRAS2	-10.1	HSPA4	-2.48	SERPINB5	-6.06

IFT74	1.9 ± 0.2	SACM1L	3.23	HSPA5	2.14	SIKE1	-3.3 ± 1.3

IGBP1	-5.6	SERPINB5	-3.59	HSPB1	-3.42	ST3GAL4	-2.79

KRT1	-2.42	SRSF9	2.37	HSPD1	4.31	TBCC	-3.7 ± 1.5

KRT13	-4.38	TBCC	-12.07	ILI2A	-2.6	TFB1M	-4.55

KRT15	-6.2 ± 1.4	TPRG1	-2.02	KIAA1524	11.9	TRAP1	2.73

KRT17	-2.37	YWHAE	-3.29	KRT15	-9.1 ± 2.5	TUBA1B	2.51

KRT18	4.0 ± 1.3	YWHAQ	-2.97	KRT17	-7.7 ± 1.8	TUBA1B	-2.11

KRT5	-11.6 ± 3.0			KRT2	-5.14	TUBBA2A	3.28

KRT6A	-1.86			KRT24	-3.75	UCHL5	-2.68

KRT6B	-3.5 ± 0.3			KRT5	-5.35	VPS39	-1.97

KRT83	-2.5			KRT6A	-4.0 ± 1.7	YWHAZ	-4.5 ± 0.5

LRPPRC	6.2 ± 1.2			LCMT1	-1.58		

Fold-change of proteins identified multiple times is indicated with the S.E.M. and the highest p-value associated with any of the spots. Proteins indicated in bold are up-regulated in 184A1 in comparison with both tumor cell lines. Proteins indicated in bold and italicized are up-regulated in both tumor cell lines in comparison with 184A1

^a ^A positive fold change indicates greater expression in the cancer cells line while a negative fold change indicates a greater expression in 184A1

### Pathway analysis and protein interaction network generation using IPA

**Figure 4:**
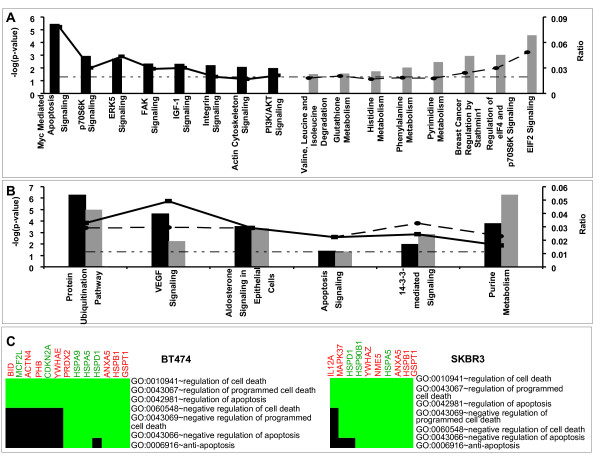
**Significant canonical pathways (*p *< 0.05) for proteins differentially expressed in BT474 (black) and SKBR3 (gray) in comparison with 184A1 cell line**. The negative of the log_10 _(*p*-value) (long dash dot dot) and ratio (number of focus molecules involved in the pathway/total number of molecules in the pathway) are plotted on the primary and secondary Y-axis respectively. Pathways for BT474 are indicated by black columns and the ratio is indicated by a solid line joined by squares. Similarly, pathways for SKBR3 are indicated by gray columns and corresponding ratio is indicated by long dashes joined by circles. (Panel A) Canonical pathways unique to each cell line. (Panel B) Canonical pathways common to both the cell lines. (Panel C) Functional clustering of gene ontology (GO) terms associated with apoptosis for differentially expressed proteins obtained using DAVID. Green region of the heat map indicates corresponding GO association positively reported and the black region indicates the GO association not reported as yet. Gene symbols colored red are down-regulated and colored green are up-regulated in tumor cell lines in comparison with 184A1.

Using the list of differentially expressed proteins for each pairwise comparison, we annotated those patterns with biological function by quantifying biological pathways that were enriched in the data sets. A statistical test, the Fisher exact test with a Benjamini-Hochberg correction, was used to assess the conditional probability of observing multiple patterns associated with a given pathway by chance alone. This statistical test was applied to all of the canonical pathways contained within the IPA library. In addition, the ratio of observed proteins relative to the total number of proteins in a pathway provided an additional metric for pathway enrichment. The results from this pathway analysis are summarized in Figure 4. Some pathways were found to be unique to each cell line (Figure 4A) and some were common to both the cell lines (Figure 4B). The most significant canonical pathway associated with differentially regulated proteins in BT474 cell line was the "Protein Ubiquitination Pathway" when ranked by significance (*p *< 5.5 × 10^-7^) with nine molecules (HSPA5, HSPA9, HSPA1L, HSPB1, HSPD1, PSMB1, PSMC2, PSMC4, and PSME1) out of a possible 274 associated with the pathway. "Myc Mediated Apoptosis Signaling" was the most significant pathway (*p *< 3.62 × 10^-6^) when ranked by ratio (0.078) with five focus molecules (BID, CDKN2A, RRAS2, YWHAE, YWHAQ) out of a possible 64 molecules being associated with the pathway. Similarly, in the SKBR3 cell line, the most significant pathway was "Purine Metabolism" when ranked by significance (*p *< 5.2 × 10^-7^) with ten molecules (ATP5B, HSPD1, MPP2, NME5, NME7, POLI, PRPS1, PRPSAP2, SEPT1, and TRAP1) out of a possible 439 being associated with the pathway. "EIF2 Signaling" was the most significant pathway (*p *< 2.91 × 10^-5^) when ranked by ratio with five focus molecules (AKT2, EIF2S1, EIF2S2, EIF3I, and PPP1CC) out of a possible 104 being associated with the pathway. Additionally, "Protein Ubiquitination Pathway" was also ranked high in SKBR3 both by significance (*p *< 1.13 × 10^-5^), as well as ratio with eight molecules (HSP90B1, HSPA4, HSPA5, HSPB1, HSPD1, PSMA3, PSMB8, UCHL5) out of a possible 274 associated with the pathway. In general, pathways associated with proteins differentially expressed in BT474 were found to be predominantly associated with cell motility and proliferation (ERK5, FAK, IGF-1, Integrin, and Actin cytoskeleton signaling). On the other hand, metabolic pathways (Glutathione, Histidine, Phenylalanine, Pyrimidine, and Purine Metabolism) formed a predominant group of canonical pathways associated with differentially expressed proteins in SKBR3.

Information obtained from IPA analysis relates canonical pathways to a group of genes, but lacks the ability to predict how a pathway is regulated differently. For instance, "apoptosis signaling" was a significant pathway in both the tumor cell lines, it remained unclear if this pathway was up- or down-regulated. Additional functional annotation was obtained using DAVID [19], which clusters highly related genes and their corresponding functional gene ontology annotation to generate a gene-term 2D heat map view (Figure 4C). Differentially expressed proteins from BT474 and SKBR3 were seen to be involved in negative regulation of apoptosis and programmed cell death.

**Figure 5:**
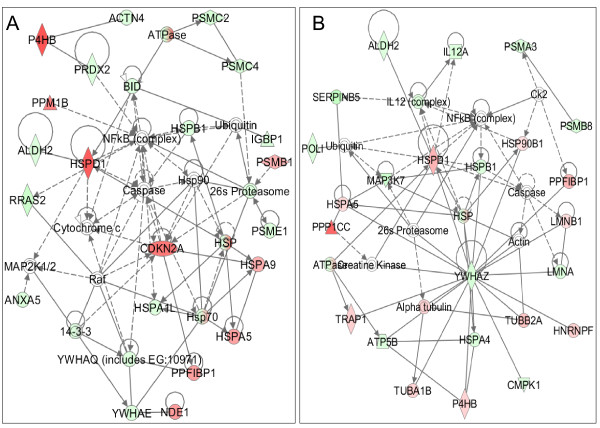
**Proteins differentially expressed in (A) BT474 and (B) SKBR3 in comparison with 184A1 were overlaid onto a global molecular network developed from information contained in the Ingenuity Knowledge Base (IKB)**. Genes or gene products are represented as nodes, and the biological relationship between two nodes is represented as an edge (line). Solid lines indicate a direct relationship and dashed lines indicate an indirect relationship between nodes. The intensity of the node color represents the degree of up- (red) or down- (green) regulation. White nodes represent the IKB molecules associated with focus genes. Network reflects (A) Cellular Function and Maintenance, Cell Death, and Protein Synthesis (*p *< 10^-48^) and (B) Cell Morphology, Cellular Function and Maintenance, and Protein Degradation (*p *< 10^-57^).

To overcome the limitation of pathway-based analysis where not all human genes have been assigned to a definitive pathway; we also tried to interpret the dataset of differentially expressed proteins in terms of a protein-interaction network. The differentially expressed proteins were uploaded and mapped to corresponding "gene objects" in the Ingenuity Pathways Knowledge Base (IPKB) in which curated prior information used is a master gene interaction network. Using the BT474 data set, ten protein interaction networks were found to be statistically significant (*p *< 0.01). Six of the ten generated networks were discarded as they had only one focus molecule in the network. Similarly, six interaction networks were generated using SKBR3 data set, out of which one network was discarded on similar grounds. The remaining networks are summarized in Additional file 4: Table S3 with the list of all proteins associated with the focus genes of the network. The most significant network with the highest number of focus molecules in BT474 had a *p*-value < 10^-48 ^with 23 focus genes mapped onto a network of 35 molecules as shown in Figure 5A and had functions associated with "Cell Death" and "Protein Synthesis". The most significant network in SKBR3 (*p *< 10^-57^) had 24 focus molecules mapped onto the protein interaction network consisting of 35 molecules (Figure 5B) and had functions associated with "Cell Morphology" and "Protein Degradation". NF-*κ*B complex formed a major hub at the centre of the network in BT474 as well as SKBR3, with a number of direct and indirect interactions with the focus molecules in the network. The intensity of the node color represents the degree of up- (red) or down-(green) regulation in tumor cell lines. Validation of these nodes is an important step as they are major gene regulators and deletion of any of these nodes may influence the inferred network.

### Validation of proteomics results using immunoblotting

We confirmed the differential expression of proteins inferred from the proteomics results using immunoblotting. Some of the proteins that were involved in the most significant pathways of both the cell lines were selected for validation. These proteins were HSPA5 and HSPD1, which are both involved in "Protein Ubiquitination Pathway", for BT474, and HSPD1 and TRAP1, which are both part of "Purine Metabolism" pathway, for SKBR3 cell line. "Apoptosis Signaling", a hallmark of cancer and a significant pathway in BT474 (*p *< 0.04) and SKBR3 (*p *< 0.05) was also selected for validation. Interestingly, different proteins were involved in apoptosis signaling in both the cell lines. In BT474, BH3 domain interacting death agonist (Bid) and RRAS2 were involved in apoptosis signaling. On the other hand, Calpain-11 (CAPN11) and LMNA were a part of apoptosis signaling pathway in SKBR3. Of these, BID and CAPN11 were selected to validate the differential expression.

**Figure 6:**
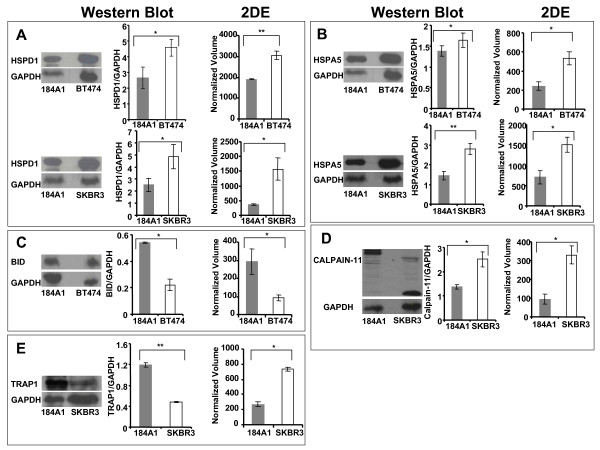
**Western blot validation of identified proteins to confirm the expression trend as inferred from 2DE**. Protein levels were normalized against GAPDH. Error bars represent S.E.M. * represents (*p *< 0.05) and ** represents (*p *< 0.01).

As summarized in Figure 6, western blotting analysis provided consistent results as compared to the gel-based proteomics results. HSPD1 (Figure 6A) was detected in three locations in the 184A1-BT474 proteomic analysis, possibly due to processing influences, and was upregulated in BT474 by 3.88 (*p *< 4.2 × 10^-4^), 1.6 (*p *< 0.04), and 4.6 (*p *< 0.02) -fold respectively. In SKBR3, HSPD1 was upregulated by a factor of 4.3 (*p *< 0.03). HSPA5 (Figure 6B) was upregulated in BT474 by 2.2-fold (*p *< 0.02) as well as in SKBR3 by 2.1-fold (*p *< 0.03). BID (Figure 6C) was found to be downregulated in BT474 by 3.1-fold (*p *< 0.05) and proteomic analysis found the 80kD isoform of CAPN11 (Figure 6D) was upregulated in SKBR3 by 3.5-fold (*p *< 0.01) in comparison with 184A1. However, the up regulation of HSP90 (Figure 6E) in SKBR3 inferred from proteomic analysis was inconsistent with immunoblotting, which revealed the protein to be upregulated in 184A1. This inconsistent observation might be a cause of antibody specificity or the semi-quantitative nature of immunoblotting given the differences in dynamic ranges of photographic quantification of chemiluminiscence and coomassie based detection methods.

## Discussion

The HER2+ cancer cell lines were selected to represent different features of breast cancer phenotypes including variations in receptor status. One of the cell lines, BT474, is derived from ER+/PR + solid invasive ductal carcinoma in the breast with a high *in vitro *invasion capability [20]. SKBR3, on the other hand is derived from pleural effusion adenocarcinoma, is negative for both, ER and PR, and exhibits a low *in vitro *invasion capability [21]. The non-tumorigenic cell line selected as a reference for comparison of differential protein expression was 184A1 as it has a lower proliferation rate as compared to other non-tumorigenic epithelial cell line, such as MCF10A [22]. The 184A1 cell line was established from a normal mammary tissue, transforming it by exposure to benzo(a)pyrene, thereby making it immortal but not malignant. In the Biology of Cancer, Robert Weinberg states that normal and cancer cells "utilize control circuitry that is almost identical. Cancer cells discover ways of making relatively minor modifications of the control machinery operating inside cells. They tweak existing controls ..." (emphasis added pg 159) [23]. Identifying these subtle differences in signaling circuitry will help understand the mechanistic basis for cancer. By focusing on a small subset of cancer cell lines, we aimed to identify these subtle differences that are lost when averaged across many different cell lines. Here we assume that the observed proteomes reflect intrinsic differences in the genetic regulatory structure that is altered in cancer. Thinking of cancer as an evolutionary process, the BT474 and SKBR3 cell lines reflect two genetic solutions to a survival problem physically encoded as the tumor microenvironment. We used proteomics to explore the characteristics of these genetic solutions. By setting a higher threshold for inclusion in the study (i.e., greater than 1.5-fold difference), we tried to focus our functional annotation on proteins that may play a role in malignant transformation. In the following paragraphs, we will highlight some of the intriguing aspects associated with differential regulation of IGF, protein ubiquitination, and apoptosis signaling pathways within these three cell models.

The pathway enrichment results suggest that flux through the IGF signaling pathway is enhanced in the BT474 relative to 184A1. Previously, we found that IGF signaling and IGF1R expression were enhanced in the BT474 relative to SKBR3 [24]. IGF signaling has been implicated in regulating epithelial-to-mesenchymal transition [25], a phenomenon associated with increased cell motility and poor treatment outcome [26,27]. The IGF signaling pathway shares common effector pathways with the insulin signaling pathway [28]. Here, the 184A1 cell line should have a higher level of insulin signaling based upon the concentration of insulin used in the tissue culture media. This is interesting as model-based inference suggests that the BT474 cell line, in contrast to both the SKBR3 and 184A1 cell lines, exhibits non-canonical activation of the IGF pathway following activation of the EGF receptor [29]. Over-expression of HER2 increases the potential for autocrine activation of the EGF receptor [30]. Validating the mechanistic explanation for these observed differences in insulin/insulin growth factor signaling is on-going.

Protein ubiquitination pathway was a highly significant pathway associated with both BT474 and SKBR3 cell lines and involved mainly proteasome subunits and heat shock proteins. As shown in Table 1, most of the proteasome subunits were downregulated (PSMA3, PSMB8, PSMC2, PSMC4, UCHL5) and heat shock proteins were upregulated (HSP90B1, HSPA5, HSPB1, HSPA9) as compared to 184A1. Down-regulation of proteasome subunits might indicate weakening of the ubiquitin-proteasome system thereby accumulating abnormal proteins that in turn might confer growth and malignant potential in these tumor cells. This might be leading to an increased turnover of heat shock proteins, which function as chaperones, to prevent the accumulation of aggregated proteins resulting from proteasome inhibition [31,32]. Heat shock proteins (HSP's) are a family of stress response proteins that play an important role in protein folding and translocation. Elevated levels of HSP's have been reported in breast cancer cells [33,34]. Overexpression of HSPA5 has been demonstrated in ER + as well as ER- tissues compared to normal tissues ranging from 1.8- to 20-fold [35]. In this study, HSPA5 was overexpressed by a factor of 2.2-fold (*p *< 0.02) in BT474 (ER+) and 2.1-fold (*p *< 0.03) in SKBR3 (ER-). Barazi et al. [36] have shown that HSPD1 can directly activate the function of α_3_β_1 _integrin, which plays an important role in tumorigenesis and metastasis of breast cancer. Additionally, Li et al. [37] have demonstrated the presence of HSPD1 at higher levels in MDA-MB-435HM (highly metastatic) cells compared to the parental cell line. They conclude that HSPD1 could be one of the potential biomarkers for breast cancer progression and metastasis.

Apoptosis signaling, another pathway common to both BT474 and SKBR3 inferred from the differential protein expression, is a process whereby cells commit to a program of organized cell death in response to external cues, such as TRAIL, or internal triggers such as DNA damage or aberrant cell cycling [38-40]. Initiation of apoptosis is regulated by the balance between pro-apoptotic proteins; such as Bax, Bak, Bad or Bid; and proteins that inhibit apoptosis, such as Bcl-2 or Bcl-XL. The balance among these proteins that promote or inhibit apoptosis determines ultimate cell fate. Quantifying gene expression is one alternative approach; however many of these apoptosis related proteins are post-translationally regulated in cancer cell lines. Bid (**B**H3-**i**nteracting **d**omain death agonist) is an apoptosis inducing protein [41], which upon activation engages the pro-survival Bcl-2-like proteins via the BH3 domain and inactivates their function [42]. Given the BH3-only proteins' ability to induce apoptosis, there has been an increasing interest in the field of cancer therapeutics to create 'BH3 mimetics' as novel anti-cancer agents [43,44]. Downregulating expression of Bid has been shown to make cells resistant to Fas-mediated apoptosis [45]. BT474 cells have also been shown to be resistant to TRA-8, an agonistic antibody to death receptor 5 that induces apoptosis in various cancer cells [46]. Preclinical studies in mouse xenografts have shown that in the presence of ispinesib, a chemotherapeutic, expression of proapoptotic proteins Bax and Bid was lower in BT474 as compared to MDA-MB-468, whereas antiapoptotic protein Bcl-XL was higher [47]. Bid, which is downregulated in BT474 cell line and involved in the apoptosis signaling pathway, might suggest that this tumor cell line acquires the hallmark of evasion of apoptosis by downregulating Bid expression.

Calpain (calcium-activated neutral protease) is another protein that plays a role in apoptosis and was differentially expressed in the SKBR3 cell line relative to 184A1. Calpain exists in two isoforms: m- and μ-calpain as 80 kDa and 30 kDa isoforms, where the prefix refers to the concentration of calcium required for activation [48]. We observed higher levels of 80 kDa isoform in the nontumorigenic 184A1 cell line and the 30 kDa isoform in the SKBR3 tumor cell line. The large and small isoforms have distinct expression patterns in human breast cancer. The larger subunit was observed at low expression levels in high grade tumors whereas the smaller subunit was observed at a high level in tumors derived from breast cancer patients with a poor prognosis and high risk for metastasis [49]. Though the exact function of calpain still remains unclear, evidence suggests a role in apoptosis because of its ability to cleave p53 [50] and mediate I*κ*B*α *proteolysis [51]. Calpain is known to regulate survival mechanisms in drug resistant cancer cells. Inhibiting the catalytic activity of calpain helps overcome resistance to TRAIL in colon cancer [52] and cisplatin in melanoma [53]. Kulkarni et al. [54] have demonstrated that calpain confers resistance to trastuzumab and apoptosis in HER2-positive breast cancer cells (SKBR3), deregulating calpain in turn deregulates activation of HER2 and PTEN/AKT1, and inversely inhibiting calpain helps in overcoming resistance to trastuzumab. It has been shown that calpains degrade Bid thereby dampening apoptotic signaling; and inversely calpain inhibition partially restores Bid levels and in turn cells sensitivity to apoptotic signaling [55]. Calpain overexpression in the SKBR3 cell line might indicate that this particular phenotype of breast cancer acquires the hallmark of apoptosis evasion via calpain mediated Bid degradation. This inference of inhibition of apoptosis is further supported by NF-κB, which forms a central inferred node in the IPA network for both cell lines. Implication of NF-κB in inhibition of apoptosis and as a therapeutic target in cancer is well known [56-59]. NF-κB was also an inferred hub of a network in another global proteomic analysis of three breast cancer cell lines (MCF7, SKBR3, MDA-MB-23) in comparison with non-transformed mammary cells (MCF10A) [60].

## Conclusion

In summary, we have inferred that multiple pathways are altered upon malignant transformation by comparing protein expression patterns of two HER2 positive breast cancer models with a transformed normal mammary cell line. These differences in functional traits reflect the genetic loci that are altered upon malignant transformation. Our data also suggest that a hallmark of cancer, evasion of apoptosis, even though common to both the HER2+ tumor models, might have different mechanisms of action. These data also motivate follow-on hypothesis-driven studies to understand how Bid and Calpain collectively regulate apoptosis, how malignant transformation alters the sensitivity of the insulin related signaling pathway to extracellular signals, and how additional signaling pathways modulate HER2 dependence. Such studies might open new perspectives for improving the efficacy of personalized medicine.

### Experimental procedures

#### Cell culture and reagents

The human breast cancer cell lines (BT474 and SKBR3) were kindly provided by Dr. Jia Luo (University of Kentucky; Lexington, KY). The nontumorigenic human breast epithelial cell line 184A1 was obtained from ATCC (Manassas, VA). Cells were grown in 75-cm^2 ^plastic tissue culture flasks (Costar Corning; Corning, NY) in a humidified incubator at 37°C and 5% (v/v) CO_2_. The BT474 cells were routinely maintained in Rosewell Park Memorial Institute (RPMI) 1640 medium (Mediatech, Inc., Herndon, VA) supplemented with 10% (v/v) heat inactivated fetal bovine serum (FBS) (Hyclone, Inc., Logan, UT), 0.3% (w/v) L-glutamine, 1% (v/v) penicillin/streptomycin (BioWhittaker, Walkersville, MD) and 10 ng/mL insulin (Sigma, St Louis, MO). SKBR3 cells were maintained in Improved Modified Eagle Medium (IMEM) Zn^2+ ^option (Invitrogen) containing 4 mM L-glutamine, 2 ml/L L-proline, 50 μg/mL gentamicin sulfate supplemented with 10% FBS (Hyclone) and 1% penicillin/streptomycin (BioWhittaker). 184A1 cell line was maintained in DMEM/Ham's F-12 (1:1) medium supplemented with 5% (v/v) horse serum (Invitrogen) in the presence of 20 ng/mL rhEGF, 10 μg/mL insulin and 0.5 μg/mL hydrocortisone (Sigma, St Louis, MO). Media was changed every 3 days and cells were passaged in a 1:3 dilution at approximately 80% confluence.

#### Sample preparation for 2-DE

Sample preparation for 2DE was done as we have previously described [24]. Briefly, cells were incubated in lysis buffer (7 M Urea, 2 M thiourea, 2% (w/v) CHAPS) for 30 min on ice and sonicated for five cycles in an ultrasonic water bath, where each sonication was performed for 30 s followed by 30 s cooling interval on ice. Cell debris were pelleted by centrifugation at 14,000 rpm for 40 min at 4°C. The supernatant was aliquoted in fresh tubes and stored at -80°C. The protein concentration was determined using CB-X™ protein assay (G Biosciences).

#### 2-D electrophoresis

For each cell line, 120 μg of cell lysate was mixed with rehydration buffer (7 M urea, 2 M thiourea, 2% CHAPS, 1% DTT, 2% IPG buffer, 0.002% bromophenol blue) and incubated for 1 h at room temperature prior to rehydration on Immobilized pH Gradient (IPG) strips pH 4-7, 7 cm, (GE Healthcare, Uppsala, Sweden) for 12 h at 25°C. Isoelectric focusing was done using Ettan IPGphor apparatus (Amersham Biosciences) for a total of 17 kVh at 50 μA per strip at 20°C in the following steps: step-n-hold at 300 V for 4 h, 1000 V gradient in 30 min, 5000 V gradient in 1 h 30 min, followed by step-n-hold at 5000 V till 17 kVh was reached. Thereafter, IPG strips were equilibrated in 75 mM Tris-HCl pH 8.8, 6 M urea, 30% (v/v) glycerol, 2% (w/v) SDS, 0.002% (w/v) bromophenol blue and 1% (w/v) DTT for 30 min. A second equilibration step was done for another 30 min by replacing the DTT with 2.5% iodoacetamide. Equilibrated strips were transferred onto 12% SDS-polyacrylamide gel. IPG strips were sealed with 0.5% (w/v) low melting point agarose in SDS running buffer containing bromophenol blue. Gels were run at 5 mA for 1 h to facilitate a gradual protein transfer from the strip onto the gel, and then at 10 mA until the dye front had run off the bottom of the gels. The coomassie stained gels were scanned using Typhoon 9400 scanner (Amersham Biosciences) at 100 μm resolution at normal sensitivity. Data were saved in .gel format using ImageQuant software (Amersham Biosciences).

#### Image analysis

The images were analyzed using REDFIN Solo software from Ludesi. Gel images were cropped to remove the boundary region without proteins. The warping was done by choosing a reference image and spot matching was facilitated by placing approximately 10 manual vectors in each quadrant of the gel to align cognate spots at corresponding locations across different gels. Normalized spot volumes were generated from the optical densities for each individual spot to the ratio of the total spot volume in each gel. Protein spots were considered to be differentially expressed if the difference between the averages of spot densities from the nontumorigenic cell line and the tumor cell lines was 1.5-fold or greater with *p *< 0.05. More than 95% of the protein spots in the analysis were present in all six gels, with few spots being present in five out of the six gels in the analysis.

#### In-gel digestion

The manually excised gel spots of interest were destained in 50-50% acetonitrile/50 mM NH_4_HCO_3 _solution, reduced in DTT (100 mM, 57°C, 45 min) and alkylated with iodoacetamide (500 mM, room temperature, 45 min) in a dark room. The gel pieces were dehydrated in acetonitrile for 10 min, were vacuum dried and rehydrated with 10 μL of digestion buffer (10 ng/μL of trypsin (Promega; Madison, WI) in 50 mM NH_4_HCO_3_) and covered with 10 μL of NH_4_HCO_3_. The samples were incubated for 16 h at 37°C to allow for complete digestion. 5% formic acid was added to stop the enzymatic digestion and the peptides were extracted in sequential steps by sonication using acetonitrile and 50% acetonitrile/0.1% TFA.

#### MALDI-TOF MS analysis

MALDI-TOF-MS system model Micromass MALDI-R (Waters^®^) was used to obtain the peptide mass fragment spectra as recommended by the manufacturer. Protein digest solutions were mixed at a 1:1 ratio with the MALDI matrix α-cyano-4-hydroxycinnamic acid (CHCA) (Sigma-Aldrich Fluka; St. Louis, MO). 2 μL of tryptic digest was applied to the MALDI plate and allowed to dry. The MALDI-TOF MS was operated in the positive ion delayed extraction reflector mode for highest resolution and mass accuracy. Peptides were ionized/desorbed with a 337-nm laser and spectra were acquired at 15 kV accelerating potential with optimized parameters. The external calibration performed using ProteoMass Peptide MALDI-MS Calibration Kit (Sigma) provided mass accuracy of 25-50 ppm. Internal calibration was performed with the monoisotopic peaks of Angiotensin II (*m/z*: 1046.5423), P_14_R (synthetic peptide) (*m/z*: 1533.8582) and adrenocorticotropic hormone (ACTH) (18-39) peptide (*m/z*: 2465.1989). Mass spectral analysis for each sample was based on the average of 1000-1200 laser shots. Peptide masses were measured from *m/z*: 800 to 3,000. The raw spectra was background subtracted, smoothed and deisotoped using ProteinLynxGlobalServer (PLGS) v2.1. The peak lists containing the *m/z *ratio and corresponding intensity values were exported to Microsoft Excel for further processing.

#### Protein identification using peptide mass fingerprinting (PMF)

Peptide mass fingerprint's (PMF) obtained from MALDI-TOF MS were used to query public protein primary sequence databases for protein identification. Monoisotopic peaks resulting from internal calibrants were removed before submitting the peak lists to the databases. Mascot database search engine v2.3.02 (http://www.matrixscience.com, Matrix Science Ltd., UK) and Expasy Aldente (version 19/03/2010) were used to query the UniProtKB/Swiss-Prot human database (Release 2010_12, 523151 sequences, 184678199 amino acids) with the following settings: peptide mass tolerance of 50 ppm, one missed cleavage site, one fixed modification of carboxymethyl cysteine, one variable modification of methionine oxidation, minimum of four peptide matches and no restrictions on protein molecular mass or isoelectric point. The combined use of two different algorithms offers an advantage of cross validating and consolidating the identification through complementary use of different packages. Aldente, for example, has an added advantage of identifying protein isoforms, a feature that is absent in Mascot. A protein was considered to be positively identified only when it was a hit using both algorithms.

#### Ingenuity pathway analysis

Differentially regulated proteins identified by 2DE and PMF were further analyzed using Ingenuity Pathway Analysis (IPA; Ingenuity Systems, Mountain View, CA; http://www.ingenuity.com). IPA was used to interpret the differentially expressed proteins in terms of an interaction network and predominant canonical pathways as described in detail earlier [24]. Briefly, a dataset containing the differentially regulated proteins, called the focus proteins, for a particular cell line was uploaded into the IPA. These focus proteins were overlaid onto a global molecular network developed from the information in the IKB. Networks of these focus proteins were then algorithmically generated by including as many focus proteins as possible and other non-focus proteins from the IKB that are needed to generate the network based on connectivity. Enriched canonical pathways were identified from the IPA library using a Fisher's exact test adjusted for multiple hypothesis testing using the Benjamini-Hochberg correction [61]. We supplemented the results of our pathway analysis using DAVID Bioinformatics Resource 6.7 [62,63], a functional annotation tool that links expression data to their gene ontology (GO) annotation and identifies clusters of common GO terms.

#### Western blotting

For western blot analysis, 10-20 μg of total cell lysate was separated by SDS-PAGE using a 12% Tris polyacrylamide gel with a 4% stacking gel at 75 V for 4 h. Proteins were transferred onto Bio Trace PVDF membrane (PALL Life Sciences; Pensacola, FL) at 42 V for 1.5 h. Blots were washed in Tris Buffered Saline (TBS) for 5 min at room temperature, blocked for 1 h in TBS + 0.1% Tween 20 (TBS/T) plus 5% dry milk at room temperature and then washed three times in TBS/T. Blots were incubated overnight at 4°C with primary antibodies specific for HSPA5 (MAB4846), HSP90 (AF3286), BID (AF846), HSP60 (AF1800) (all from R&D Systems Inc., Minneapolis, MN), and Calpain11 (ab28227) (Abcam Inc., Cambridge, MA). The next day, blots were washed three times in TBS/T, incubated for 1 h at room temperature with anti-biotin (Cell Signaling Technology, Inc., Danvers, MA, 7727) and either an anti-mouse IgG-HRP (HAF007), anti-rabbit IgG-HRP (HAF008), or anti-goat IgG-HRP (HAF017) (all from R&D Systems Inc.). Finally, the blots were washed three times in TBS/T, developed using LumiGLO reagent (Cell Signaling Technology, Inc., Danvers, MA, 7003) and bands were visualized on KODAK Biomax light film (Fisher Scientific). Densitometric analysis was performed using ImageJ software (National Institute of Health) and protein levels were normalized to GAPDH (sc-25778) (Santa Cruz Biotechnology Inc., Santa Cruz, CA) protein levels for each sample.

#### Statistics

Unless otherwise indicated, the comparison between groups for western blots was performed using a standard Student's *t*-test assuming equal variance among samples. A *p*-value of 0.05 was considered significant and data is expressed as mean ± standard error of independent experiments.

## Competing interests

The authors declare that they have no competing interests.

## Authors' contributions

YK was responsible for the 2-DE, MALDI-TOF MS, PMF, IPA analysis, and immunoblotting. DK conceived of the study, participated in its design, analyzed data and coordinated its execution. All authors drafted, read and approved the final manuscript.

## Supplementary Material

Additional file 1 **Figure S1**. Three biological replicates of the cellular proteome of (A) 184A1, (B) BT474 and (C) SKBR3 resolved on 7 cm IPG strip 4-7. Click here for file

Additional file 2 **Table S1**. Identification summary of the differentially expressed proteins in 184A1-BT474 comparison showing the rank and score from two different algorithms used to search the protein database. Worksheet 2 shows the sequence coverage, peptide matches, number of mass values searched and the RMS error. Click here for file

Additional file 3 **Table S2**. Identification summary of the differentially expressed proteins in 184A1-SKBR3 comparison showing the rank and score from two different algorithms used to search the protein database. Worksheet 2 shows the sequence coverage, peptide matches, number of mass values searched and the RMS error. Click here for file

Additional file 4 **Table S3**. Summary of IPA generated networks for proteins differentially expressed in BT474 (four networks) and SKBR3 (five networks). Molecules in bold are the focus molecules associated with the dataset and the score is the *p*-value associated with the network. Click here for file
