# Supplementary material for: Protein-based identification of quantitative trait loci associated with malignant transformation in two HER2+ cellular models of breast cancer
Source: Proteome Sci. 2012 Feb 22;10:11. doi: 10.1186/1477-5956-10-11 (PMC3305585; doi:10.1186/1477-5956-10-11)
Supplement: Additional file 1 — Figure S1. Three biological replicates of the cellular proteome of (A) 184A1, (B) BT474 and (C) SKBR3 resolved on 7 cm IPG strip 4-7. [file 1477-5956-10-11-S1.PDF]

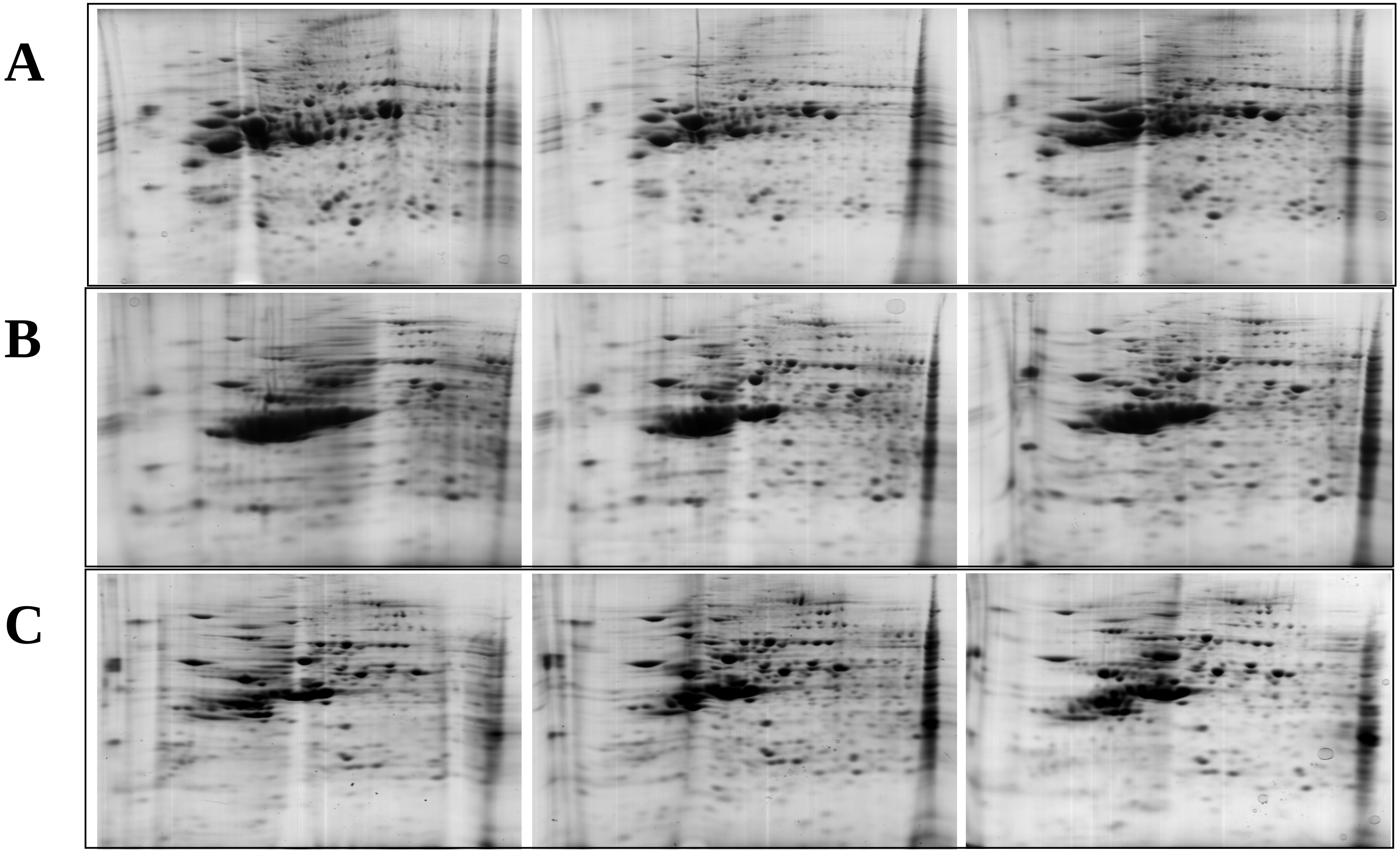

Supporting Figure S1. Three biological replicates of the cellular proteome of (A) 184A1, (B) BT-474 and (C) SK-BR-3 resolved on 7cm IPG strip 4-7.
