# Supplementary material for: Protein-based identification of quantitative trait loci associated with malignant transformation in two HER2+ cellular models of breast cancer
Source: Proteome Sci. 2012 Feb 22;10:11. doi: 10.1186/1477-5956-10-11 (PMC3305585; doi:10.1186/1477-5956-10-11)
Supplement: Additional file 4 — Table S3. Summary of IPA generated networks for proteins differentially expressed in BT474 (four networks) and SKBR3 (five networks). Molecules in bold are the focus molecules associated with the dataset and the score is the p-value associated with the network. [file 1477-5956-10-11-S4.DOC]

Protein interaction networks for proteins differentially expressed in:

**A. BT474**

| ID | Molecules in network | Score | Focus molecules | Top functions |
| --- | --- | --- | --- | --- |
| 1 | 14-3-3, 26s Proteasome, **ACTN4**, **ALDH2**, **ANXA5**, ATPase, **BID**, Caspase, **CDKN2A**, Cytochrome c, HSP, Hsp70, Hsp90, **HSPA5**, **HSPA9**, **HSPA1L**, **HSPB1**, **HSPD1**, **IGBP1**, MAP2K1/2, **NDE1**, NFkB (complex), **P4HB**, **PPFIBP1**, **PPM1B**, **PRDX2**, **PSMB1**, **PSMC2**, **PSMC4**, **PSME1**, Raf, **RRAS2**, Ubiquitin, **YWHAE**, **YWHAQ (includes EG:10971)** | 48 | 23 | Cellular Function and Maintenance, Cell Death, Protein Synthesis |
| 2 | **ALCAM**, **ANXA8L2**, **CAPZB**, CDCA7L, **DPYSL4**, DPYSL5, **EEF1G**, **EIF2B3**, GSTA1, GSTA5, **GSTP1**, HEBP2, HNF4A, **IFT74**, INS1, IRF6, KLF5, **LRPPRC**, MAP2K4, MDH1, MIR124-1 (includes EG:406907), MYC, NFE2, OGFOD2, ONECUT1, **PIH1D1**, **PMM2**, **POLR3E**, **PPFIBP1**, **PSMC4**, **RAB27B**, retinoic acid, **RGN**, **RNPEPL1**, SUCLA2 | 37 | 17 | Cell Morphology, Connective Tissue Development and Function, Drug Metabolism |
| 3 | **ACTB**, Akt, **ALCAM**, CD6, **CSK**, ERK, ERK1/2, F Actin, Focal adhesion kinase, **FSTL1**, GSTA2, GSTA5, GSTM1, GSTM2, **GSTP1**, Histone h4, IKK (complex), Insulin, Jnk, **LYN**, mannitol, Mapk, **MCF2L**, MIR1, P38 MAPK, **PCYT1B**, **PDE4D**, PI3K (complex), Pkc(s), PRKCH, **RAB3A**, Ras, **SERPINB5**, **SRSF9**, TAOK2 (includes EG:9344) | 24 | 12 | Drug Metabolism, Glutathione Depletion In Liver, Lipid Metabolism |
| 4 | ACTN1, AXL, **BCAR3**, BCR, CCL5, EGF, GAS6, GH1, **GNA14**, GSN, HAX1, Jnk, LCK, MAPK8, MYD88, PDIA3, **PHB (includes EG:5245)**, phosphatidylinositol-3,4,5-triphosphate, phosphatidylinositol-3-phosphate, PIK3CA, Pkc(s), PRKCA, PRKCD, PRKCE, PRKCZ, PTK2, Rac, Ras, ROCK2, **SACM1L**, SET, SOS1, sphingosine-1-phosphate, STAT3, SYK | 6 | 4 | Cellular Movement, Cellular Growth and Proliferation, Tissue Development |

**B. SKBR3**

| ID | Molecules in network | Score | Focus molecules | Top functions |
| --- | --- | --- | --- | --- |
| 1 | 26s Proteasome, Actin, **ALDH2**, Alpha tubulin, **ATP5B**, ATPase, Caspase, Ck2, **CMPK1**, Creatine Kinase, **HNRNPF**, HSP, **HSP90B1**, **HSPA4**, **HSPA5**, **HSPB1**, **HSPD1**, IL12 (complex), **IL12A**, **LMNA**, **LMNB1**, **MAP3K7**, NFkB (complex), **P4HB**, **POLI**, **PPFIBP1**, **PPP1CC**, **PSMA3**, **PSMB8**, **SERPINB5**, **TRAP1**, **TUBA1B**, **TUBB2A**, Ubiquitin, **YWHAZ** | 57 | 24 | Cell Morphology, Cellular Function and Maintenance, Protein Degradation |
| 2 | AKAP13, ATF6B, Ctnna, **CTNNA3**, CTNNB1, DHX8, EWSR1, GTF3C2, GTF3C3, **GTF3C4**, GTF3C5, HNF4A, **MRPS35**, NFYB, **NME7**, **PAF1**, POLR3C, POLR3F, POLRMT, **PRPS1**, PRPSAP1, **PRPSAP2**, **RAB37**, ribose-phosphate diphosphokinase, **RMND1**, RPL31, TBP, **TFB1M**, TFPT, TXNL1, **UCHL5**, USP42, VIPAR, **VPS39** | 23 | 12 | Nucleic Acid Metabolism, Small Molecule Biochemistry, Cellular Assembly and Organization |
| 3 | ADRB1, AKAP5, AKAP12, amino acids, Calpain, **CAPN11**, **EEF2K**, EIF2B4, GRB2, **GRK4**, GSTA1, GSTM2, **GSTP1**, INS1, Insulin, **KIAA1524**, **LCMT1**, MAP2, MAP2K7, MTORC1, MYC, **NQO2**, **OPA1**, PPME1, PRKACB, PRKAG1, PRKCG, PRKCH, RAGE, retinoic acid, **SEMG2**, SEMG1 (includes EG:6406), St3gal, **ST3GAL4**, TNF | 19 | 10 | Protein Synthesis, Cell Morphology, Connective Tissue Development and Function |
| 4 | ACAP1, ADRB1, AKAP5, AKAP11, AKAP13, Akt, **BPNT1**, CARD11, CAV3, DLG1, **EIF3I (includes EG:8668)**, **GDI2**, GRM5, **GSTP1**, I kappa b kinase, IFNB1, Il18r, KCNQ1, **MPP2**, NDUFA13, **NME5**, NR3C1, PRKACB, PRKAG1, PRKAR1A, **PRKAR2A**, PSAP, RAB11A, **RAB3IL1**, **SEPT1**, **SIKE1**, TJP2, TOLLIP, TRAF6, TRAF3IP2 | 19 | 10 | Post-Translational Modification, Infection Mechanism, Cell Morphology |
| 5 | ADRB1, Akt, **AKT2**, **ANXA5**, **ANXA6**, Ap1, **APOA1**, **EIF2S1**, **EIF2S2**, ERK, ERK1/2, **GNB1L**, GSTA1, GSTA2, GSTA5, GSTM1, GSTM2, **GSTP1**, IKK (complex), IL17RD, Insulin, Interferon alpha, Jnk, LDL, MAFK, mannitol, P38 MAPK, Pkc(s), PPM1L, **PRDX6**, PRKCH, Ras, Sod, TAOK2 (includes EG:9344), VRK2 | 16 | 9 | Drug Metabolism, Glutathione Depletion In Liver, Lipid Metabolism |
